# *Chlorophytum borivilianum* Polysaccharide Fraction Provokes the Immune Function and Disease Resistance of* Labeo rohita* against* Aeromonas hydrophila*

**DOI:** 10.1155/2015/256510

**Published:** 2015-11-15

**Authors:** Sib Sankar Giri, Shib Sankar Sen, Cheng Chi, Hyoun Joong Kim, Saekil Yun, Se Chang Park, V. Sukumaran

**Affiliations:** ^1^Laboratory of Aquatic Biomedicine, College of Veterinary Medicine and Research Institute for Veterinary Science, Seoul National University, Seoul 151742, Republic of Korea; ^2^School of Life Sciences, Jawaharlal Nehru University, New Delhi 110067, India; ^3^Department of Biotechnology, Periyar Maniammai University, Thanjavur, Tamil Nadu 613403, India

## Abstract

The present study aimed to investigate the effects of* Chlorophytum borivilianum* polysaccharide (CBP), as a dietary supplement administered at varying concentrations with feed (basal diet), on various cytokine-related responses in* Labeo rohita* fingerlings. Immune parameters and immune-related gene expressions were measured at 3rd, 4th, and 5th week after feeding. The results revealed that dietary administration of CBP at 0.2% and 0.4% for 4 weeks significantly upregulated serum lysozyme and phagocytic activity. Complement C3 and respiratory burst activity (RBA) were significantly higher after 4 weeks of CBP feeding. The immune related genes* IL-8*,* IL-1β*,* TNF-α*, and* iNOS* were downregulated (*P* < 0.05) in groups with 0.2% and 0.4% CBP supplemented diets at week 4. Expression of anti-inflammatory cytokines (*IL-10* and* TGF-β*) was also downregulated (*P* < 0.5) after 4 weeks of feeding with 0.2% to 0.8% CBP. However, five weeks of CBP administration had no significant effect on immune gene expression, except* TNF-α* and* IL-8*. Fish fed with 0.4% CBP for 4 weeks showed maximum resistance against* Aeromonas hydrophila* (73.3% survival) compared to control. From these results, we recommend that CBP administration at 0.4% for 4 weeks could effectively improve immune response and disease resistance in* L. rohita*.

## 1. Introduction

Indian aquaculture production consists mainly (~70%) of three major carps,* Labeo rohita, Catla catla, *and* Cirrhinus mrigala *[1]. Intense culture practices generate potential environmental stressors that lead to high susceptibility of captive fish species to various diseases, including viral, bacterial, fungal, and parasitic infections. Moreover, commercial aquaculture has been hampered by economic loss as a result of infectious diseases [2]. Typically, antibiotics, vaccines, and chemotherapeutics are used for disease control in aquaculture. However, treatment with antibiotics leads to the development of drug resistant pathogens, environmental hazards, and food safety problems [3]. Further, single vaccine application is only effective against one type of pathogen, and the vaccination of juvenile fish is labor intensive and expensive [4]. Therefore, it is imperative to develop natural or eco-friendly therapeutics to ensure the sustainability of aquaculture. Since fish depend primarily on innate immune function rather than on specific immunity, immunostimulants play a major role in disease resistance by enhancing innate immunity [5]. Additionally, several physiological parameters, such as respiratory burst activity (RBA), nitric oxide synthase, lysozyme, bactericidal activity, and antibody response, serve as good immunological indicators in fish.

Cytokines, protein mediators produced by immune cells, are responsible for host innate defense mechanisms. Several cytokine and immune-related genes have been identified and characterized in fish [2, 6–8]. Interleukins (ILs), tumor necrosis factors (TNFs), transforming growth factor (TGF), chemokines, and interferons (IFNs) are types of cytokines which require cytokine receptors. These cytokines are reported to have proinflammatory, anti-inflammatory, and pathogen-killing activities [9]. Hence, understanding differential cytokine expression can enable the development of immunostimulants for aquaculture [10]. Further, obtained information has the added advantage of identifying potential indirect immunological markers for aquatic species.

Several studies have investigated the enhancement of growth, immunity, and disease resistance in fish through dietary administration of immunostimulants. Stimulation of fish immunity through dietary immunostimulants is of high interest for commercial aquaculture [8]. Several herbal preparations have been reported to enhance the immune responses in fish [2, 4, 7, 11–15]. For example,* Rehmannia glutinosa *root products enhanced the growth and immune parameters of* Cyprinus carpio* [2]. Seed of* Achyranthes aspera *enhanced the immune responses in* Catla catla* [7]. The root of* Withania somnifera *improved the disease resistance of* Labeo rohita* against* Aeromonas hydrophila* [13]. Recently, we found that* Psidium guajava *leaves as feed additive could increase the growth performance, disease resistance, and cytokine gene expression of* Labeo rohita* [14]. Herbal therapeutic products are less expensive and have greater accuracy compared with chemotherapeutic agents, providing a potential solution for the problems facing aquaculture today [15].

*Chlorophytum borivilianum* L. (Liliaceae) is a traditional rare medicinal herb in India and considered “white gold” or “divya aushad” in Indian systems of medicine. Its root tubers are widely used for various therapeutic applications. The traditional uses of* C. borivilianum* tubers against diseases such as diabetes mellitus, dysuria, diarrhea, and dysentery are well documented in the literature [16, 17]. Several studies have reported that* C. borivilianum* possesses various pharmacological properties such as antimicrobial [18], anti-inflammatory [19], antioxidant [20, 21], antistress [22], antidiabetic [23], immunomodulatory [16, 24], antiulcer [25], and anticancer effects [26]. Major phytochemical constituents isolated from the roots of* C. borivilianum *include steroidal saponins, fructans and fructooligosaccharides, phenolic compounds, acetylated mannans, and proteins [16, 18, 21, 22]. Polysaccharide fractions derived from the root tubers of* C. borivilianum *have been shown to exhibit modulation of human natural killer (NK) cell activity, humoral response to sheep red blood cells (SRBCs), and enhanced immunoglobulin G levels in rats [16]. Moreover, polysaccharides from natural resources are a class of macromolecules that can efficiently affect the immune system and therefore have much importance in basic and applied research [6]. Hence, we hypothesize that polysaccharide fractions of* C. borivilianum* may boost the immune response of teleosts. However, very few studies have investigated the medicinal properties of* C. borivilianum*.

To date, no investigation has been conducted to exploit the potential efficacy of* C. borivilianum* as a feed additive in modulating fish growth and immune response. The present study was designed to investigate the effects of dietary supplementation of a polysaccharide extracted from* C. borivilianum* root on the growth performance, immune parameters, and expression of several immune-related genes in* L. rohita*. Further, the efficacy of this polysaccharide in the disease resistance of* L. rohita* was also investigated through challenge study.

## 2. Materials and Methods

### 2.1. Diet Preparation

Dried roots of* Chlorophytum borivilianum* were collected from a safed musli farm, Indore (Madhya Pradesh), India. The roots were dried in sunlight and coarsely powdered for extraction. The powdered roots were subjected to hot water extraction following the method described by Thakur et al. [16]. The purified polysaccharide fraction was lyophilized and stored at −20°C for further use.

A basal diet comprising 39% groundnut oil cake, 34% rice bran, 20% soybean meal, 5% fish meal, and 2% mineral and vitamin mixture (every 250 g of mineral-vitamin mixture provided vitamin A, 500000 IU; vitamin D3, 100000 IU; vitamin B2, 0.2 g; vitamin E, 75 units; vitamin K, 0.1 g; calcium pantothenate, 0.25 g; nicotinamide, 0.1 g; vitamin B12, 0.6 mg; choline chloride, 15 g; calcium, 75 g; manganese, 2.75 g; iodine, 0.1 g; iron, 0.75 g; zinc, 1.5 g; copper, 0.2 g; and cobalt, 0.045 g) was prepared [28]. Proximate analysis of basal diet performed according to AOAC method [29] revealed 37.3% protein, 8.6% lipid, and 12.1% ash. The basal diet was considered as control diet. The* C. borivilianum* root polysaccharide (CBP) was supplemented into basal diet at five levels: 0% (basal diet), 0.1% (D1), 0.2% (D2), 0.4% (D3), and 0.8% (D4). All the ingredients were blended thoroughly in a mixture, pelletized, air-dried, ground, and sieved into proper pellet size. All feeds were stored at 4°C until use.

### 2.2. Cytotoxicity of CBP

Rat macrophage cells (ATCC CRL-2192) were routinely cultured in DMEM (Dulbecco's Modified Eagle Medium) supplemented with 10% horse serum (HS), penicillin G (500 U/mL), streptomycin (5000 *μ*g/mL) and 3.5 mg/mL of D-glucose at 37°C, and 5% CO_2 _in a humidified incubator (Sanyo, Japan). To maintain exponential growth, cells were seeded at 1 × 10^5^ cells/mL and passaged every 5-6 days. Chlorambucil (Sigma-Aldrich, USA) at concentration 600 *μ*g/mL was used as positive control [16]. Cell viability was assessed using ATPlite kit (Perkin Elmer, USA). CBP was assayed at various concentrations starting at 10 mg/mL. Test was performed in triplicate. For this, 50 *μ*L of CBP sample was added to each well along with 50 *μ*L of cell suspension. The cells and samples were incubated at 37°C for 24 h in a humidified (5% CO_2_) incubator (Sanyo, Japan). Measurement of cell proliferation was performed using Micro Beta 1450 plate reader (Perkin Elmer, USA) following ATPlite kit instructions.

### 2.3. Test Fish and Experimental Protocol

*Labeo rohita* fingerlings (mean bodyweight 10.3 ± 0.07 g) were acclimatised to laboratory conditions for 2 weeks in 500-litre plastic quarantine tanks at 26 ± 2°C. Fish were fed with basal diet during the acclimatisation period. About 20% of the water in all tanks was exchanged daily and 100% of the water was exchanged once a week. Basic physiochemical parameters of the water were measured every week [30]. The O_2 _and ammonia concentrations were ranged from 6.1 to 7.3 mg L^−1^ and 0.03 to 0.06 ppm, respectively, and pH was ranged from 7.0 to 8.0 throughout the study period.

Fish were randomly stocked in fifteen tanks with a stocking density of 30 fish per tank with triplicates per dietary treatment. Capacity of each tank was 200 litres. All the fish were fed with one of the five diets (basal diet, D1, D2, D3, and D4) for 5 weeks at the rate 2–4% of body weight and fed twice a day (9.00 and 17.00). Any uneaten feed left was removed after one hour of feeding and dried, weighed, and finally subtracted from the total amount of supplied diets to calculate the actual feed intake. The water quality was checked regularly.

### 2.4. Growth Performance

Ten fish were randomly collected from each tank at end of 3rd, 4th, and 5th week of feeding for batch weighing. Growth performances and survival rate of fish were calculated using the following formula:

(1) Weight  gain  (WG;g/fish)=Wt−W0,Specific  growth  rate  (SGR)=100×ln⁡Wt−ln⁡W0t,Feed  conversion  ratio  (FCR)=FIWt−W0,Survival  %=100×final  no.  fishinitial  no.  of  fish,

where *W*_*t*_ and *W*_0_ were final and initial weight of fish, respectively; “*t*” is the duration of feeding (in days); FI is feed intake.

### 2.5. Immune Parameters

Five fish were randomly collected from each tank at end of 3rd, 4th, and 5th week of feeding to measure the immune parameters. Thus, a total of 15 fish (3 × 5 = 15) were collected in each group for immunological assays. Blood samples were collected by caudal venipuncture using a 1 mL syringe after anesthetizing the fish with diluted MS222 (Sigma-Aldrich, USA). The blood samples were transferred into microcentrifuge tubes. After collection, blood was centrifuged at 3500 g at 4°C for 10 min and obtained serum was stored at −20°C.

#### 2.5.1. Serum Lysozyme Activity

Lysozyme activity (LA) was measured according to the method described by Ellis [31]. One unit of lysozyme activity was defined as the amount of enzyme producing a decrease in absorbance of 0.001 min^−1 ^mL^−1^ serum.

#### 2.5.2. Complement C3 Assay

The serum complement C3 level was assayed using a C3 kit (Biocompare, CA, USA). C3 level analysis included measurement of the increase in turbidity after immunity response of C3 and its increased antibody [32]. Results of C3 activity were expressed as C3 mg mL^−1^.

#### 2.5.3. Phagocytic Assay

*Aeromonas hydrophila *MTCC-1739 was cultured in tryptic soy agar medium (Sigma-Aldrich) for 24 h at 37°C. The cell number was adjusted to 5 × 10^6^ CFU/mL. The phagocytic activity of the blood leukocyte was determined according to the method of Cai et al. [33]. The phagocytic activity (%) was calculated as using the formula:

(2) $$\begin{array}{c} \left[\;100\times \left(\;\text{phagocytic leucocytes}\;\right){\left(\;\text{total leucocytes}\;\right)}^{-1}\;\right]. \end{array}$$

#### 2.5.4. Respiratory Burst Activity (RBA)

The RBA of phagocytes was measured using the nitroblue tetrazolium (NBT, Sigma-Aldrich) assay following the method of Secombes [34] with previously described modifications [35]. Color development was measured at 630 nm with a spectrophotometer (Bio-Rad, India).

### 2.6. Expressions of Immune-Related Genes

Head kidney was collected from 15 fish per group, frozen in liquid nitrogen, and stored at −80°C. Total RNA was extracted from head kidney using TRIZOL (Invitrogen, USA) according to the manufacturer's instructions. RNA concentration and purity were quantified using a spectrophotometer (NanoDrop 2000c, Thermo scientific), and the quality was checked using a 1% agarose gel containing 0.5 *μ*g mL^−1^ ethidium bromide. RNA was reverse-transcribed to cDNA using a SuperScript cDNA synthesis kit (Life Technologies, USA), following the manufacturer's instructions. Real-time PCR analysis of IL-8, IL-1*β*, IL-10, iNOS, TNF-*α*, and TGF-*β* and a housekeeping gene (*β*-actin) was carried out with CFX96 Real-Time PCR (Bio-Rad, Laboratories, Inc.) following standard protocols with the primer sequences and thermocycling conditions indicated in Table 1. To verify the accuracy of each amplicon, melt curve analysis was performed after amplification. All samples were run in parallel with the housekeeping gene in order to normalize cDNA loading. Gene expression results were analyzed using the 2^−ΔΔCT^ method after verifying the primers amplified with an efficiency of approximately 100% [36], and data for all treatment groups were compared to those of the control groups.

### 2.7. Challenge Test

The seven-day lethal dose 50 (LD_50_) for* A. hydrophila* MTCC-1739 was 10^7^ CFU/mL as determined earlier in our laboratory [28]. At the end of the feeding trial, ten fish from each tank (3 × 10 = 30 fish per group) were picked out for challenge test. The fish were injected i.p. with 100 *μ*L of phosphate buffer saline (PBS) containing 1 × 10^7^ live* A. hydrophila*. The challenged fish were kept under observation for 2 weeks and fed a basal diet. The mortality of fish in each tank was observed over the course of 14 days.

### 2.8. Statistical Analysis

One way analysis of variance (ANOVA) was used to analyze the data. Multiple comparisons were performed with Tukey's test to analyze the differences between treatments. All statistical analyses were performed using the OriginPro software (version 8; OriginLab Corporation, Northampton, USA). The level of significance was set at *P* < 0.05 and the results are expressed as mean ± S.E.M.

## 3. Results

### 3.1. Cytotoxicity Evaluation

Evaluation of cytotoxicity and determination of IC_50 _was carried out on mouse macrophage cell line. The IC_50_ values for chlorambucil and CBP were 32.62 *μ*g/mL and 891.17 *μ*g/mL, respectively. This high IC_50 _value of CBP confirmed its nontoxic nature.

### 3.2. Growth Performance

Dietary administration of CBP to captive carp increased growth parameters such as percent weight gain (PWG) and specific growth rate (SGR) although the increment was not significant at any point in time (data not shown). However, a significant reduction in FCR value was observed after 4 weeks of feeding with 0.4% CBP (group D3). No mortality was recorded in any group during the experimental feeding.

### 3.3. Immunological Parameters

**Figure 1:**
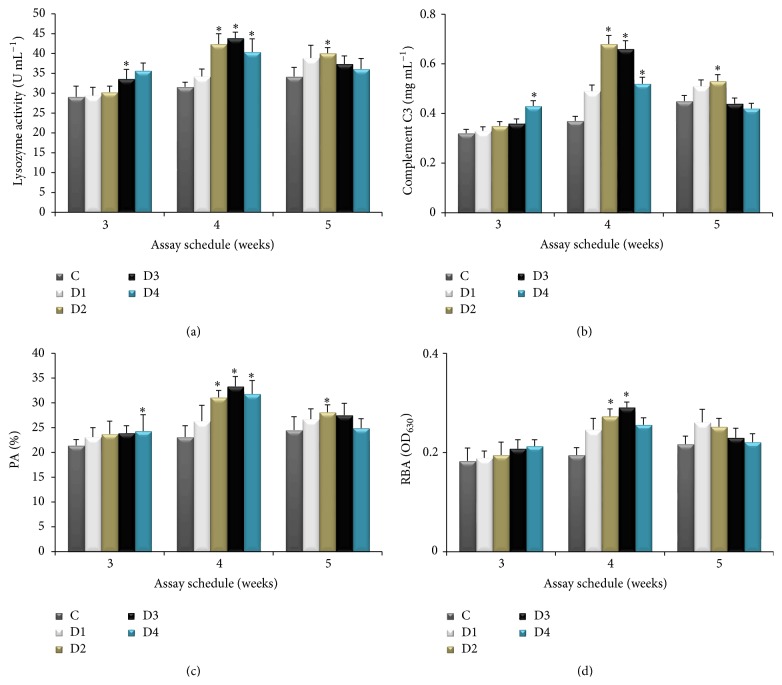
Serum lysozyme activity, complement C3, phagocytic activity (PA), and respiratory burst activity (RBA) of* Labeo rohita* (*n* = 15) at the end of weeks 3, 4, and 5 of feeding with a graded level of CBP. Bars represent the mean ± SEM and asterisks represent levels of significant differences compared to the control (*P* < 0.05). Note: C (basal diet); D1 (basal diet + 0.1% CBP); D2 (basal diet + 0.2% CBP); D3 (basal diet + 0.3% CBP); D4 (basal diet + 0.4% CBP).

Results of the immunological parameters are shown in Figure 1. Serum lysozyme activity (Figure 1(a)) showed maximum peak at 4 weeks of feeding regime. Fish fed 0.2–0.8% CBP supplemented diets for 4 weeks had significantly higher lysozyme activity and the highest was in the group (D3) fed 0.4% CBP. CBP had no significant effect of lysozyme activity at 5 weeks of feeding, except in the group fed 0.2% CBP.

In the case of complement C3 activity, a significant increment was observed in dietary supplementation of 0.2–0.8% CBP for 4 weeks and the highest C3 activity was recorded in the group fed 0.4% CBP (Figure 1(b)). However, dietary CBP administration for 5 weeks had no significant effect on C3 activity, except in the group fed 0.2% CBP.

CBP administration for 3 weeks had no significant effect on phagocytic activity (PA) (Figure 1(c)) and respiratory bursts activity (RBA) (Figure 1(d)) in rohu. However, CBP administration at 0.2–0.8% (D2–D4) for 4 weeks enhanced PA and RBA compared to the control (*P* < 0.05), and highest activities were observed in the group (D3) fed 0.4% CBP. After 5 weeks of CBP feeding, significantly higher PA and RBA were observed in groups D2 and D1, respectively.

### 3.4. Expression of Immune Related Genes

**Figure 2:**
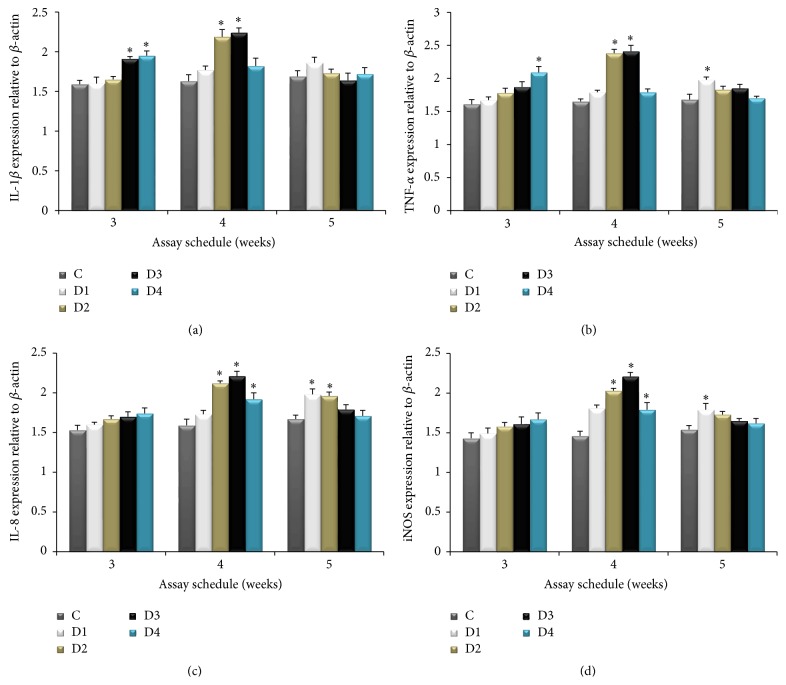
The relative mRNA expression of IL8, IL-1*β*, iNOS, and TNF-*α* in the head kidney of* Labeo rohita* at the end of weeks 3, 4, and 5 of feeding with graded level of CBP. Bars represent the mean ± SEM and asterisks represent levels of significant differences compared to the control (*P* < 0.05). Note: C (basal diet); D1 (basal diet + 0.1% CBP); D2 (basal diet + 0.2% CBP); D3 (basal diet + 0.3% CBP); D4 (basal diet + 0.4% CBP).

**Figure 3:**
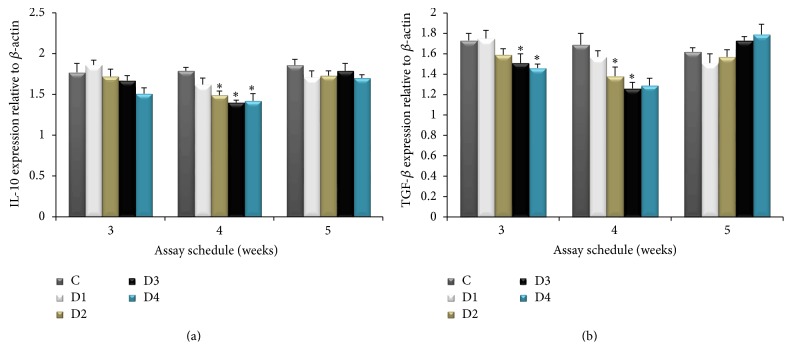
The relative mRNA expression of IL-10 and TGF-*β* in the head kidney of* Labeo rohita* at the end of weeks 3, 4, and 5 after administration of varying concentrations CBP. Bars represent the mean ± SEM (*n* = 15) and asterisks represent levels of significant differences compared to the control (*P* < 0.05). Note: C (basal diet); D1 (basal diet + 0.1% CBP); D2 (basal diet + 0.2% CBP); D3 (basal diet + 0.3% CBP); D4 (basal diet + 0.4% CBP).

The expression profiles of six immune-related genes in the head kidney of fish were investigated at the end of 3, 4, and 5 weeks of CBP administration. The expression levels of four proinflammatory cytokines (IL-8, IL-1*β*, iNOS, and TNF-*α*) were upregulated after 4 weeks of feeding (Figure 2), whereas two anti-inflammatory cytokines (IL-10 and TGF-*β*) were downregulated after four weeks of feeding with CBP (Figure 3).

The IL-1*β* expression was upregulated at 0.4% and 0.8% CBP administration for 3 weeks (*P* < 0.05), while after 4 weeks of CBP supplementation, higher expression (*P* < 0.05) was observed in groups administered 0.2% and 0.4% CBP (Figure 2(a)). TNF-*α* expression was higher (*P* < 0.05) in the group fed 0.8% CBP after 3 weeks (Figure 2(b)). However, maximum (*P* < 0.05) TNF-*α* expression was achieved in groups administered 0.2% and 0.4% CBP groups after 4 weeks of feeding. Administration of 0.2–0.8% CBP for 4 weeks provoked (*P* < 0.05) IL-8 expression in the head kidney of rohu and this trend continued until the end of the trial in the groups administered 0.2% and 0.4% CBP (Figure 2(c)). Dietary administration of CBP upregulated iNOS expression (*P* < 0.05) at 4 weeks of feeding compared to the control (Figure 2(d)), and the highest expression levels were in the group (D3) administered 0.4% CBP. After 5 weeks, only the group administered 0.1% CBP exhibited a higher iNOS expression level. In general, prolonged supplementation of CBP attenuated the expected levels of IL-1*β*, TNF-*α*, and iNOS expression.

Effects of CBP on IL-10 and TGF-*β* mRNA expression are shown in Figure 3. Dietary administration of CBP attenuated the expected rate of expression of IL-10 and significant attenuation was observed at 4 weeks of 0.2–0.8% CBP administration (Figure 3(a)). However, 5 weeks of CBP supplementation had no significant effect on IL-10 and TGF-*β* transcription. A significant attenuation of TGF-*β* expression was observed in the group fed 0.4% and 0.8% CBP at 3 weeks of feeding, but 4 weeks of CBP administration attenuated (*P* < 0.05) TGF-*β* expression (Figure 3(b)) in all experimental groups.

### 3.5. Challenge Test

**Figure 4:**
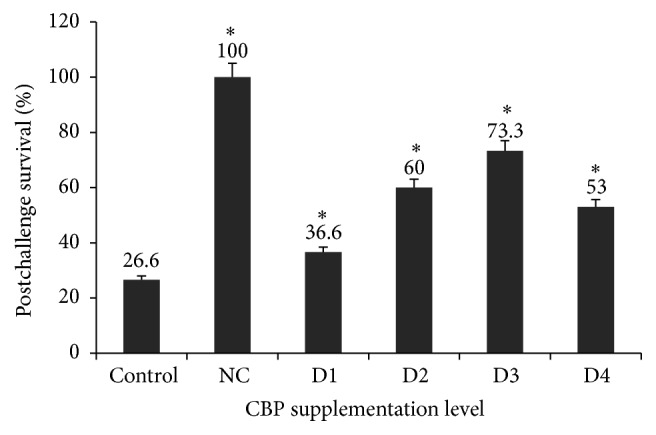
Effect of dietary CBP administration on the survival of* Labeo rohita* (*n* = 30) after challenge with* A. hydrophila*. Postchallenge survival (percent) in each dietary group is indicated at the top of each column. Bars represent the mean ± SEM and asterisks represent levels of significant differences compared to the control (*P* < 0.05). Note: NC (negative control); C (basal diet); D1 (basal diet + 0.1% CBP); D2 (basal diet + 0.2% CBP); D3 (basal diet + 0.3% CBP); D4 (basal diet + 0.4% CBP).

Results of the challenge study revealed that fish fed 0.4% CBP (group D3) exhibited highest postchallenge survival (73.3%), followed by 0.2% CBP (group D2) fed group (60% survival), and 0.8% CBP (group D4) supplementation (53.3% survival) (Figure 4). Fish on the control diet without CBP exhibited the lowest postchallenge survival rate (26.6%).

## 4. Discussion

*Chlorophytum borivilianum* is renowned for its ability to boost immune function [24]. In the present study, we report the effects of* C. borivilianum* polysaccharide (CBP) on immune response and disease resistance of rohu (*L. rohita)*. We found that CBP had no significant effect on growth performance, but administration of 0.4% CBP resulted in lower (*P* < 0.05) FCR. The significant reduction in FCR suggests that the fish utilize nutrients more efficiently when their diet is supplemented with CBP.

Lysozyme, present in the mucus, plasma, and other body fluids of most fish species, plays an important role in mediating protection against microbial invasion [37]. Our study showed that fish fed with 0.2–0.8% CBP supplemented diets for 4 weeks had significantly higher serum lysozyme activity. Our results are in agreement with earlier investigations which report the increment of serum lysozyme activity through dietary administration of* Ficus carica *polysaccharide [8] and* Astragalus *polysaccharides [6] in grass carp and common carp, respectively. We observed that higher levels (0.4%–0.8%) of CBP supplementation for 5 weeks had no significant effect on LA in the present study. This is likely because higher doses of immunostimulants for longer durations lead to immunosuppression [11].

The complement system is an important component of the innate immune response and plays a critical role in alerting, as well as in clearing, potential pathogens in the host [38]. In the present study, CBP supplementation significantly stimulated the complement C3 level up to 4 weeks, and thereafter it declined. Our results are in agreement with the results of Yang et al. [8]. Complement activation is usually beneficial to fish, but constant activation could cause side effects and immune suppression to the host [39]. Hence, enhancement of serum complement C3 in CBP fed groups up to 4 weeks after administration may be beneficial to fish.

Phagocytosis is responsible for early activation of the inflammatory response before antibody production [40]. Phagocytosis-associated respiratory burst activity (RBA) is considered to be a vital indicator of nonspecific defense in fish [41]. Respiratory bursts are produced by phagocytes in order to attack invasive pathogens during phagocytosis and it is being used to evaluate the defense ability against pathogens [41]. In this study, enhanced PA and RBA (*P* < 0.05) were observed in groups D2, D3, and D4 (administered 0.2–0.8% CBP) after 4 weeks and highest was in D3 (i.e., 0.4% CBP) group. Similarly, dietary administration of guava leaves enhanced (*P* < 0.05) the phagocytic activity of blood leucocytes in* L. rohita* [14]. In* Cyprinus carpio* and* Larimichthys crocea*, RBA of phagocytic cells has been significantly enhanced after feeding with a mixture of two herbal extracts [41]. Dietary* A. aspera* seeds enhanced RBA in carp* Catla catla* [7]. The increased activities of serum lysozyme, complement C3, PA, and RBA in the present study reveal that dietary supplementation of CBP has a significant role in enhancing the immune response in rohu. However, increased levels of this dietary supplementation over longer periods decreased lysozyme and complement C3, RBA, and phagocytic activities, suggesting that higher levels of CBP supplementation may depress fish immunity. This is most likely because higher doses of immunostimulants administered over longer periods often lead to immunosuppression [14].

Beside the enhancement of immune responses in rohu, our study also provides evidence that CBP can effectively regulate the expressions of certain cytokine-related genes such as Il-8, IL-1*β*, TNF-*α*, iNOS, IL-10, and TGF-*β*. To the best our knowledge, this is the first report about the effect of CBP on cytokine gene expressions in fish. Therefore, the present results of the gene expression study are compared with earlier studies reported about the effect of herbal preparations on cytokine gene expressions in fish.

IL-1*β* is a proinflammatory cytokine that affects almost every cell type in concert with another proinflammatory cytokine, TNF [42]. Both IL-1*β* and TNF-*α* are cytokines that induce an inflammatory response. IL-1*β* plays an important role in host response to microbial invasion, tissue injury, and immunological reactions, while TNF-*α* is crucial for diverse cellular responses like cell proliferation, differentiation, and induction of other cytokines [43]. In the present study, fish groups fed with 0.2% and 0.4% CBP supplemented diets showed significantly higher IL-8, IL-1*β*, and TNF-*α* expression than the control; however, their expression abundance exhibited differences in the same organ of fish fed different levels of CBP. Many studies have demonstrated that herbal immunostimulants can induce proinflammatory cytokines. Grass carp fed with* F. carica *polysaccharide for three weeks exhibited strong upregulation of IL-1*β* and TNF-*α* expression [8]. The oral administration of* Spirulina platensis *augmented the expression of IL-1*β* and TNF-*α* genes in common carp [44].

Production of iNOS is a well-known immunoregulatory factor in fish challenged with various pathogens [45]. We found that dietary administration of CBP for 4-week changes (*P* < 0.05) iNOS transcription in rohu. Transcription of iNOS was upregulated in the head kidney of common carp following* Rehmannia glutinosa* supplementation for 80 days [2]. Recently, we demonstrated that dietary administration of guava leaves at 0.5%–1.5% attenuated the expected expression of iNOS in head kidney, intestine, and hepatopancreas of rohu [14], which is in contrast to the result of the present study. However, the upregulation of IL-8, IL-1*β*, and iNOS gene expression in the present study may increase production of reactive-nitrogen intermediates that can damage pathogens [46].

IL-10 is a multifunctional cytokine with immunosuppressive and cytokine synthesis inhibitory functions [47]. The prime function of IL-10 is to counteract proinflammatory cytokines such as IL-1*β* and TNF-*α* to prevent tissue damage [48]. Another cytokine, TGF-*β*, generally inhibits B and T cell proliferation and differentiation, antagonizes proinflammatory cytokines such as IL-1*β*, TNF-*α*, and IFN-*γ*, and suppresses the expression of IL-1*β* and IL-2 receptors. It also directly targets effector T cells and Treg cells to ensure self-tolerance [49]. In the present study, IL-10 and TGF-*β* expression was downregulated in the head kidney of rohu fed 0.2−0.8% CBP for 4 weeks. Similarly, IL-10 expression was downregulated in the kidney of* Catla catla* fed a 1.0%* A. aspera *supplemented diet [7]. Common carp fed a diet supplemented with* R. glutinosa* had lower IL-10 and TGF-*β* expression levels in the kidney, spleen, and intestine [2]. Oral administration of spirulina in common carp induces a decrease in IL-10 gene expression, which is similar to the decrease that we observed [44]. Contrary to our results, Zhang et al. [50] reported that bath delivery of immunostimulants can induce higher expression of TGF-*β* in rainbow trout fry. In a recent study, we found that rohu fed diet supplemented with guava leaves exhibited downregulation in IL-10 and TGF-*β* gene transcription [14]. Therefore, we suggest that downregulation of the anti-inflammatory cytokines IL-10 and TGF-*β* may favor the enhanced expression of proinflammatory cytokines in CBP-fed fish.

After challenge with* A. hydrophila*, the highest postchallenge survival rate (73.3%) was exhibited by the fish group fed a 0.4% CBP supplemented diet, while the second-highest survival (63.3%) was exhibited by the group supplemented with 0.2% CBP. The enhanced immune parameters (e.g.,LA, ACP, PA, and RBA), stimulation in proinflammatory cytokines, and declines in anti-inflammatory cytokines in rohu administered 0.4% CBP might be associated with improved resistance against* A. hydrophila* and the resulting higher postchallenge survival percentages. Further, the group administered 0.8% CBP did not exhibit the highest postchallenge survival, and this may be associated with the results of immune responses and immune gene expression. Recently, Wang et al. [2] reported that dietary administration of* R. glutinosa* enhanced the postchallenge survival of common carp against* A. hydrophila* infection. Oral administration of azadirachtin increased the resistance of gold fish* Carassius auratus* against* A. hydrophila* infection [15]. Grass carp fed with* F. carcica* polysaccharide had remarkably higher resistance against* Flavobacterium columnare* [8]. However, the effect of* C. borivilianum* polysaccharide oral administration should be investigated further to elucidate the molecular mechanism by which disease resistance is conferred.

## 5. Conclusions

This study has provided the first evidence for how* C. borivilianum* provokes immune responses in fish. The administration of CBP influences both innate and humoral immunity in rohu and provokes the expression of immune related genes. Among the different dietary level of CBP, administration at 0.4% (D3) for 4 weeks efficiently upregulated (*P* < 0.05) the immune parameters and expressions of proinflammatory cytokines (IL-8, IL-1*β*, TNF-*α*, and iNOS) and downregulated the expressions anti-inflammatory cytokines (TGF-*β* and IL-10 gene). As CBP administration at 0.4% was shown to provide extraordinary protection in fish against pathogen challenge, CBP at 0.4% for four weeks can be used as a feed additive in carp breeding to improve immunity and disease resistance. However, further studies must be carried out to explore the active compounds in CBP and their detailed mechanisms in stimulating immunity.

## Figures and Tables

**Table 1:** Real-time primer sequences and thermocycling conditions.

Target gene	Primer sequence (5′ to 3′)	Thermocycling conditions	Reference/accession number
IL-1*β*	ATCTTGGAGAATGTGATCGAAGAG GATACGTTTTTGATCCTCAAGTGTGAAG	95°C 30 s, 40 cycles of 95°C 5 s, 61.5°C 30 s, and 72°C 30 s	AM932525

IL-10	AAGGAGGCCAGTGGCTCTGT CCTGAAGAAGAGGCTCTGT	95°C 30 s, 40 cycles of 95°C 5 s, 61.1°C 30 s, and 72°C 30 s	AB010701

iNOS	GGAGGTACGTCTGCGAGGAGGCT CCAGCGCTGCAAACCTATCATCCA	95°C 30 s, 40 cycles of 95°C 5 s, 61.1°C 30 s, and 72°C 30 s	AM932526

TNF*α*	CCAGGCTTTCACTTCACG GCCATAGGAATCGGAGTAG	95°C 30 s, 40 cycles of 95°C 5 s, 61.1°C 30 s, and 72°C 30 s	FN543477

TGF-*β*	ACGCTTTATTCCCAACCAAA GAAATCCTTGCTCTGCCTCA	95°C 30 s, 40 cycles of 95°C 5 s, 60.5°C 30 s, and 72°C 30 s	AF136947

IL-8	GGGTGTAGATCCACGCTGT AGGGTGCAGTAGGGTCCA	95°C 30 s, 40 cycles of 95°C 5 s, 60.5°C 30 s, and 72°C 30 s	Self-design

*β*-actin	AGACCACCTTCAACTCCATCATG TCCGATCCAGACAGAGTATTTACGC	95°C 30 s, 40 cycles of 95°C 5 s, 60.5°C 30 s, and 72°C 30 s	[27]
